# The Metacognitive and Neurocognitive Signatures of Test Methods in Academic Listening

**DOI:** 10.3389/fpsyg.2022.930075

**Published:** 2022-06-10

**Authors:** Jiayu Zhai, Vahid Aryadoust

**Affiliations:** ^1^School of English Studies, Sichuan International Studies University, Chongqing, China; ^2^National Institute of Education, Nanyang Technological University, Singapore, Singapore

**Keywords:** listening comprehension assessment, non-invasive neurotechnologies, eye-tracking, functional near-infrared spectroscopy, metacognitive awareness

## Abstract

This study aims to investigate whether and how test takers’ academic listening test performance is predicted by their metacognitive and neurocognitive process under different test methods conditions. Eighty test takers completed two tests consisting of while-listening performance (WLP) and post-listening performance (PLP) test methods. Their metacognitive awareness was measured by the Metacognitive Awareness Listening Questionnaire (MALQ), and gaze behavior and brain activation were measured by an eye-tracker and functional near-infrared spectroscopy (fNIRS), respectively. The results of automatic linear modeling indicated that WLP and PLP test performances were predicted by different factors. The predictors of WLP test performance included two metacognitive awareness measures (i.e., person knowledge and mental translation) and fixation duration. In contrast, the predictors of the PLP performance comprised two metacognitive awareness measures (i.e., mental translation and directed attention), visit counts, and importantly, three brain activity measures: the dmPFC measure in the answering phase, IFG measure in the listening phase, and IFG measure in the answering phase. Implications of these findings for language assessment are discussed.

## Introduction

Listening is a complex and dynamic process which lies at “the heart of language learning” (Vandergrift, 2007, p. 191). Some researchers view listening as a language skill inseparable from other language skills such as reading and writing, which makes listening an even more complex activity to unpack and examine (Bachman and Palmer, 1996; Kumaravadivelu, 2003; Weideman, 2021). Due to its complexity and dynamicity, listening is never easy to assess. As Alderson and Bachman (2001) claimed, listening assessment is “one of the least understood, least developed and yet one of the most important areas of language testing and assessment” (p. x). Listening comprehension, the core component of the construct of listening, is generally assumed to consist of two cognitive processes: bottom-up processing and top-down processing (e.g., Buck, 2001; Field, 2004). Bottom-up or literal processing involves the decoding of the smallest linguistic units and rebuilding them progressively into larger units, whereas top-down or inferential processing consists of the incorporation of listeners’ prior knowledge in the generation of mental representation of the message they hear (Kintsch, 1998; Field, 2004; Aryadoust, 2019a).

Besides the complex cognitive processes involved, listening also encompasses affective, behavioral, and neurological dimensions (Rost, 2016; Worthington and Bodie, 2018; Aryadoust et al., 2020, 2022). Previous research on these listening dimensions has identified two groups of factors that are essential indicators of listening test performance. First, test-specific factors refer to test method effects that impact test takers’ listening performance at the behavioral as well as neurocognitive levels (Aryadoust et al., 2020, 2022), while listener-specific factors concern the effect of listeners’ features and characteristics on their assessment performance and are indicated by metacognitive awareness (e.g., Goh and Hu, 2014; Vandergrift and Baker, 2015), gaze behaviors (e.g., Suvorov, 2015; Aryadoust, 2019b; Batty, 2020; Holzknecht et al., 2020), and brain activity (e.g., Aryadoust et al., 2020). To date, however, there has been a dearth of research on the collective effect of these factors on listening assessment performance measured by test scores. A comprehensive investigation of test-specific (i.e., listening test methods) and listener-related factors (i.e., metacognitive awareness, gaze behavior, and brain activity) will allow us to generate an extensive and precise account of listening test performance by synergizing neurocognitive, behavioral, and stimuli-specific factors.

### Metacognitive Awareness in L2 Listening Assessment

Metacognition is defined as the “high-order cognition about cognition” (Veenman et al., 2006, p. 5). According to Flavell (1976), it concerns “one’s knowledge concerning one’s own cognitive processes and products or anything related to them” (p.232.) and “active monitoring, consequent regulation and orchestration of these processes in relation to the cognitive objects or data on which they bear, usually in the service of some concrete goal or objective” (p. 232).

Flavell’s (1976, 1979) conceptualization of metacognition was adapted by Vandergrift and Goh (2012) in their metacognitive framework of L2 listening. Vandergrift and Goh (2012) defined metacognitive awareness as listeners’ state of consciousness about their cognitive process when they are involved in a learning activity. Vandergrift and Goh (2012) further identified three components of metacognitive awareness: metacognitive knowledge, metacognitive experience, and strategy use. In line with Flavell (1979), metacognitive knowledge refers to knowledge of the factors affecting cognitive processes in L2 listening and is subcategorized into three types: person, task, and strategy. Person knowledge pertains to the self-appraisal of a listener, specifically, knowledge of the affective factors facilitating listening comprehension and development, while task knowledge is knowledge of the purposes and demands of listening tasks. Finally, strategy knowledge refers to the declarative knowledge of strategy use to accomplish listening tasks and develop listening proficiency in the long run. Metacognitive experience in L2 listening is described as sensing, such as a feeling or thought about one’s own listening cognitive process when completing listening tasks. Strategy use, the proceduralization of declarative strategy knowledge, is when a listener applies appropriate strategies to complete listening tasks. Metacognitive knowledge and strategy use can be recalled in metacognitive experiences, which in turn helps to develop metacognitive knowledge of person, task, and strategy knowledge, and promotes effective application of strategies.

The significance of metacognitive awareness in L2/academic listening has been established in both theoretical frameworks and empirical studies. Specifically, metacognitive awareness is a crucial part of listening ability and a major construct for listening assessment in the framework describing listening ability in L2 listening assessment (Buck, 2001), a regulator of the whole listening cognitive processes in the cognitive model of listening comprehension (Vandergrift and Goh, 2012), a key cognitive person factor in the systems model of listening (Vandergrift and Goh, 2012), and an indispensable part of strategic competence in the integrated cognitive theory of listening comprehension (Aryadoust, 2019a). In empirical studies, metacognitive awareness has been verified as an important predictor of L2 listening performance (Vandergrift et al., 2006; Goh and Hu, 2014; Vandergrift and Baker, 2015; Wang and Treffers-Daller, 2017; Wallace, 2020; Sok and Shin, 2021). Although the share of variance that metacognitive awareness accounts for is wide-ranging (from 4 to 22%) across these studies, largely due to the participants’ differences (e.g., age and L1), the predictive role of metacognitive awareness in L2 listening performance has been undeniably established by these studies. The findings of these studies are in line with Hulstijn’s (2015) core-peripheral model of language which postulates that metacognitive awareness is a peripheral component of language. In other words, it has a “less-linguistic or non-linguistic nature, [… and includes] strategic or metacognitive abilities related to performing listening, speaking, reading or writing tasks” (Hulstijn, 2011, p. 242), whereas the core component pertains to the linguistic knowledge and the speed processing of that knowledge. It is expected that test takers’ language performance on listening tasks be correlated more with core components and less with peripheral components (Hulstijn, 2011).

### Gaze Behavior in Listening Assessment

Test takers’ gaze behaviors can be measured by eye-tracking, which is the real-time registration of eye movement *via* a series of hardware and software (Batty, 2020). The rationale behind using eye-tracking in language research is that eye movement or gaze behavior is viewed as a window into cognition (Spivey et al., 2009; Conklin et al., 2018), an assumption known as the eye-mind hypothesis (Just and Carpenter, 1980). Eye-tracking technology in academic listening assessment has been used only recently. Researchers have used this technology to investigate the viewing patterns of visual information in video-based listening tests (Suvorov, 2015; Batty, 2020), the item reading and answering patterns in response to different item formats before and during audio text listening (Aryadoust, 2019b), the effect of the spatial location of key answers in four-option MCQs on listeners’ viewing behaviors, test performance, and item difficulty (Holzknecht et al., 2020), the test method effects and cognitive load in listening tests (Aryadoust et al., 2022), and the strategy use in listening tests as self-reported by test takers compared with that measured by eye-tracking (Low and Aryadoust, 2021). These studies support the use of eye-tracking for a variety of research purposes in listening assessment.

### Neurocognition in Listening Assessment

Compared with eye-tracking, neuroimaging is quite underutilized in (academic) language assessment research. When a region of the brain is triggered by external stimuli (e.g., language input), it starts to absorb more energy and oxygen which is transferred by oxygenated hemoglobin (Pfeifer et al., 2018). There are several neuroimaging techniques that can be used in listening assessment research, notably functional near-infrared spectroscopy (fNIRS), which is a non-invasive and user-friendly optical neuroimaging technology to measure changes in hemodynamics and oxygenation in the brain cortex (Scholkmann et al., 2014; Tak and Ye, 2014; Pfeifer et al., 2018; Sulpizio et al., 2018).

The brain cortex in the left hemisphere plays an essential role in language (and listening) comprehension. Literal processing, including phonological decoding, word recognition, semantic retrieval, and syntactic processing, is mediated by the left inferior frontal gyrus (IFG) and posterior middle temporal gyrus (pMTG), whereas inferential processing, mainly incorporating prior knowledge to make inferences based on audio inputs, is supported by the dorsomedial prefrontal cortex (dmPFC). Specifically, IFG has been found to maintain local coherence through literal processing (Buchweitz et al., 2014) and regulate semantic and syntactic processing at the local level (Keller et al., 2001; Jobard et al., 2007; Rogalsky et al., 2008; Binder et al., 2009; Friederici, 2011; Whitney et al., 2011). Relatedly, pMTG has been shown to co-function with IFG to co-facilitate semantic processing in regulating literal comprehension (Hallam et al., 2018) and support phonological, lexical, semantic, and syntactic processing (Cabeza and Nyberg, 2000; Michael et al., 2001; Wagner et al., 2001; Whitney et al., 2011).

In addition, dmPFC has been associated with inference-making in Miller and Cohen’s (2001) study where the prefrontal cortex was identified as a top-down mechanism that performs judgmenttasks and in Binder et al.’s (2009) study that reported dmPFC was associated with inferential, goal-directed retrieval of semantic information and high-level, global comprehension processes. The association between language comprehension and activation of brain cortices provides theoretical justification for using fNIRS to access test takers’ neurocognitive mechanisms in the present study.

Despite the well-established utility of fNIRS in investigating language processing, it is under-utilized in listening assessment research with few published studies identified (Aryadoust et al., 2020, 2022; Lee et al., 2020). By examining test takers’ brain activation in the dmPFC, IFG, and pMTG *via* fNIRS, these studies found that listeners’ brain activation differed not only under different test conditions compared with natural sound conditions (Lee et al., 2020) but also across different listening test methods (Aryadoust et al., 2020, 2022). Notably, the findings suggest that listeners’ test performance at the behavioral level (i.e., test scores) may not correlate with their neurocognitive processes and that (i) no significant difference in listeners’ test performance was found in listening tests which induce significantly different brain activation (Aryadoust et al., 2020) and (ii) listeners’ better test performance was found in listening tests which impose a lower cognitive load on them (Aryadoust et al., 2022). These studies support the multidimensional nature of listening, suggesting that listening assessments should not only focus on listeners’ test scores at the behavioral level but also investigate their neurocognitive processes, especially under different listening test methods.

### Test Method Effects in Listening Assessment

Test method in this study refers to the presentation format of audio texts and test items in a listening test. From this perspective, there are two test methods in L2 listening assessment: while-listening performance (WLP) and post-listening test (PLP) performance tests (Aryadoust, 2012). In a WLP test such as the listening sections of the International English Language Testing System (IELTS), test items are presented concurrently with the audio text and test takers are required to read and answer the test items while listening. By contrast, in a PLP test, like listening sections of the Test of English as a Foreign Language internet-based test (TOFEL iBT), test takers listen to the audio text, take notes, and subsequently read and answer the test items.

Previous studies have shown that the WLP and PLP test methods elicit different strategy use (Field, 2009), involve significantly different brain activation (Aryadoust et al., 2020), and induce different cognitive load on test takers (Field, 2009; Aryadoust et al., 2022). Specifically, test takers are inclined to using the test-wise strategy of keyword matching in WLP tests, that is, matching keywords and phrases against those presented in the audio text (Field, 2009). This is because the simultaneous presence of test items and the audio text, as required by the WLP test method, might have engaged test takers to source for cues in the test items (Field, 2009). Furthermore, based on test takers’ retrospective reports, Field (2009) claimed that WLP tests imposed a higher cognitive load on test takers because they needed to multitask in reading and completing test items and listening to the audio text. However, this finding contrasts with a recent study conducted by Aryadoust et al. (2022) which found the dmPFC and IFG were significantly less activated in WLP tests than PLP tests, suggesting that WLP tests imposed a lower cognitive load on test takers than PLP tests. This finding was further buttressed by the eye-tracking evidence in the study.

Different test methods also elicit different use of metacognitive strategies in listening tests. In a recent study, In’nami and Koizumi (2022) found that planning and evaluation are associated with listening comprehension only in the WLP test where multiple-choice options were presented before and throughout the test. However, the other metacognitive strategies (i.e., person knowledge, mental translation, directed attention, and problem solving) were associated with both WLP and PLP test formats.

## The Present Study

Although some researchers have highlighted the multidimensionality of listening processes (e.g., Rost, 2016; Worthington and Bodie, 2018), empirical studies pertaining to this assumption are scant in the listening assessment literature, with previous studies mostly examining the postulated dimensions of the listening construct separately. In a recent study, Aryadoust et al. (2022) examined test method effects on the cognitive load of listening test takers, using gaze behavior and brain activity. While the study found that different test methods yield different amounts of cognitive load, it did not investigate the role of metacognitive awareness or whether there is a relationship between listener-related factors in predicting listening and test scores.

In the present study, we sought existing gaze behavior, neurogaming, and test data from Aryadoust et al. (2022). In addition, we further included five variables representing the five dimensions of metacognitive awareness in listening (Vandergrift et al., 2006), comprising directed attention, mental translation, planning and evaluation, problem solving, and person knowledge. Using this collection of data, we aimed to investigate the relationship between listening test scores and test takers’ metacognitive awareness, gaze measures, and brain activity across WLP and PLP test methods. To our knowledge, there is no study in the listening assessment literature examining the cumulative effect of listener-related factors (i.e., test takers’ gaze behavior, brain activity, and metacognitive awareness) on test performance across test methods.

To address the preceding research gap, this study aims to explore whether test takers’ behavioral performance measured by test scores can be predicted by test takers’ neurophysiological process (measured by gaze behavior and brain activation) and self-appraisal of metacognitive awareness under different listening test methods (i.e., WLP and PLP tests). Based on the research objective, the research question of the present study is as follows: What is the relationship between gaze behaviors, brain activation, and metacognitive awareness of listening test takers and their test performance under the WLP and PLP test conditions?

## Materials and Methods

### Data Source

The study population in this study is defined as listening test takers. The sampling method was convenience sampling due to practicality. The participants were recruited in an English-medium university in [masked location] *via* posters and social media platforms. Eighty self-reported neurotypical participants (*M* = 24.14, SD = 4.03 years; 35 females and 45 males) were recruited. Of these, English was the first language for 48 participants (M_WLP_ = 9.00, SD_WLP_ = 1.22; M_PLP_ = 8.04, SD_PLP_ = 1.69) and the second language for 32 participants (M_WLP_ = 7.81, SD_WLP_ = 2.18; M_PLP_ = 6.67, SD_PLP_ = 1.89). This study was approved by the Internal Review Board (IRB) of the university. The participants’ informed consent was obtained before data collection.

### Research Design

**FIGURE 1:**
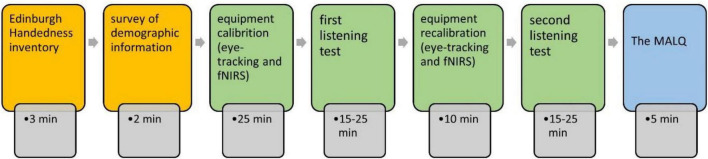
Research design. fNIRS, functional near-infrared spectroscopy; MALQ, metacognitive awareness listening questionnaire.

This study was conducted in a laboratory over 80 sessions. Each session involved one participant and lasted for approximately 85 min (see Figure 1). The participants’ handedness and demographic information was first collected. Next, two computer-mediated listening tests under the WLP and PLP test conditions were administered. A single-blinded and randomized crossover design was adopted to avoid the sequence effect (Liu, 2010). During the tests, the participants’ gaze behavior and brain activation data were collected by an eye-tracker and fNIRS, respectively (the setup information is in Table 1). The participants’ metacognitive awareness was measured after the listening tests.

**TABLE 1:** The setup of the eye-tracker and fNIRS used in the present study.

Eye-tracker	fNIRS
A stand-alone infrared eye tracker (Tobii X3-120) was mounted to a 23-inch desktop monitor.	Participants wore a customized aluminum fNIRS headcap to minimize the near-infrared light interference from the eye-tracker.
The monitor was connected to a primary laptop with the Tobii Pro Studio package.	The headcap was connected to a portable fNIRS system.
Participants sat 65 cm in front of the monitor.	Eight pairs of light-emitting sources and detectors were placed at approximately 1.5 cm from each other on the headcap to measure the activation of three brain areas (dmPFC, IFG, and pMTG) in the left-hemisphere.
Participants’ gaze behaviors were record at 120 Hz.	Participants’ hemodynamics were measured at 7.81 Hz.
Automatic calibration was performed before each listening test.	Automatic calibration was performed before each listening test.
A c-pod was used to synchronize the eye-tracking and neuroimaging data from SuperLab Version 5.0.5 (Cedrus Corporation, 2015) to NIRStar Version 15-0 (NiRx Medical Technologies LLC, 2016b)	

*See Aryadoust et al. (2022) for details.*

The data of this study partly originates from Aryadoust et al. (2022). However, the present study differs from Aryadoust et al. (2022) in three different ways: (i) we use metacognitive awareness measures in the present study alongside the eye and brain measures used in Aryadoust et al. (2022); (ii) while Aryadoust et al.’s (2022) study evaluated the difference in the cognitive load caused by test methods, the present study investigates whether and how the listening test scores can be predicted by metacognitive awareness, gaze behavior, and brain activity across two test methods; and (iii) Aryadoust et al. (2022) applied RANCOVA, Mann-Whitney U tests, and Wilcoxon signed rank tests to examine cognitive load, while we aim to apply the automatic linear modeling (ALM) to investigate how listening test scores are predicted by metacognitive awareness, gaze, and brain activity measures across test methods.

### Instruments

#### The Metacognitive Awareness Listening Questionnaire

The metacognitive awareness listening questionnaire (MALQ) is intended to measure the metacognitive awareness of L2 listeners by eliciting their self-perceived metacognitive strategy use and metacognitive knowledge (Vandergrift et al., 2006). The MALQ adopts a six-point Likert scale ranging from “strongly disagree” to “strongly agree” and comprises 21 randomly ordered items assessing five dimensions of L2 listeners’ metacognitive awareness: directed attention, mental translation, planning and evaluation, problem solving, and person knowledge. The five dimensions of metacognitive awareness constitute five subscales of the MALQ, which were psychometrically validated using Winsteps Version 4.7.1 (Linacre, 2020) and had high Rasch item reliability (Table 2).

**TABLE 2:** Rasch item reliability of the four listening tests and the five subscales of the MALQ.

	WLP-1	WLP-2	PLP- 1	PLP- 2	PK	PE	DA	MT	PS
Item reliability	0.77	0.52	0.76	0.73	0.91	0.91	0.95	0.98	0.91

*DA, directed attention; MALQ, Metacognitive Awareness Listening Questionnaire; MT, mental translation; PE, planning and evaluation; PK, person knowledge; PLP, post-listening performance; PS, problem solving; WLP, while-listening performance.*

#### Listening Tests

The listening tests were comprised of the Lectures (Section 4) of two forms of the IELTS listening tests, hereafter called IELTS-1 and IELTS-2. The two lectures shared similar linguistic features computed using Coh-Matrix (McNamara et al., 2014). The participants were required to listen to the audio texts and complete each test item. A dichotomous scale (0, 1) and full credit were used for scoring. The WLP and PLP versions of each lecture were created, forming four listening tests (WLP-1, PLP-1, WLP-2, and PLP-2). The tests were psychometrically validated using Winsteps Version 4.7.1 (Linacre, 2020) and had medium to high Rasch item reliability ranging from 0.52 to 0.77 (Table 2).

During the tests, the gaze behavior and brain activation data were collected. In line with previous eye-tracking studies (Aryadoust, 2019b; Aryadoust et al., 2022), fixations and visits were examined in this study and were measured in durations (at least 300 ms long) and counts. Therefore, four eye-tracking variables were generated: fixation duration, fixation count, visit duration, and visit count. Raw brain activation data collected using the NIRSport device were exported to NIRSLab Version 2016.06 (NIRx Medical Technologies LLC, 2016a) for pre-processing. This study only analyzed the oxygenated hemoglobin (HbO) values of which wavelength data were transformed into numerical data, i.e., beta value, as HbO values best represent brain activation (Strangman et al., 2002). The beta values in the same brain area were summed and averaged, generating an average beta value for each brain area (dmPFC, IFG, and pMTG) under three conditions (i.e., WLP, the audio texts listening phase of PLP (PLP-Audio), and the answering questions phase of PLP (PLP-Question), respectively.

### Data Analysis

Automatic linear modeling (ALM) was used as the data analysis method of this study. ALM can predict a continuous-scale target (dependent variable) based on linear relationships between the target variable and one or more predictors (IBM, 2018). ALM is a novel form of linear regression available in SPSS since version 19. Traditional linear regression is subject to several limitations, such as no capability to conduct all-possible-subsets (best subsets) regression, limited optimality statistics for variable section, and no ability to automatic process outliers and missing data (Yang, 2013). ALM is an improvement over the traditional technique, particularly due to its affordance for automatic variable selection and automatic data preparation.

**FIGURE 2:**
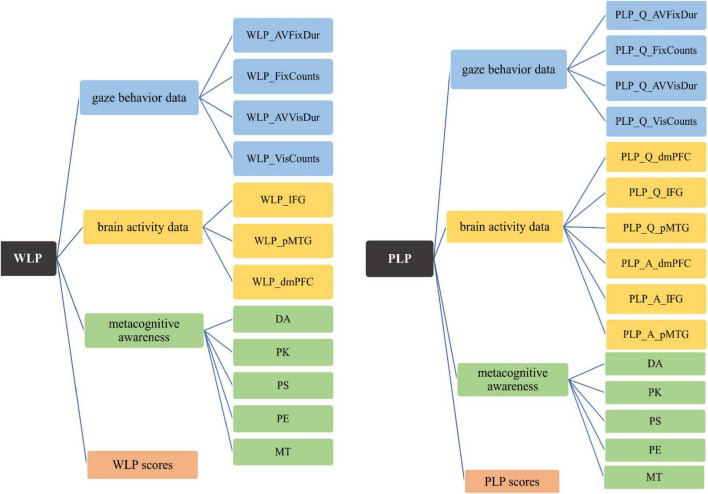
The variables used in the current study. A, the audio texts listening phase; AVFixDur, average fixation duration; AVVisDur, average visit duration; DA, directed attention; dmPFC, dorsomedial prefrontal cortex; FixCounts, fixation counts; IFG, inferior frontal gyrus; MT, mental translation; PE, planning and evaluation; PK, person knowledge; PLP, post-listening performance; pMTG, posterior middle temporal gyrus; PS, problem solving; Q, the answering phase; VisCounts, visit counts; WLP, while-listening performance.

As regards the parameters setting in ALM in this study, the default “create a standard model” was selected in main objective setting, because this method can build a single model to predict the target variable using the predictors and is easier to interpret compared with the other options (IBM, 2018). Next, automatic data preparation (ADP) was set. As a major advantage of ALM, ADP can help the data to be cleaned and prepared for use (Yang, 2013) and maximize the predictive power of the model (IBM, 2018). As for model selection, ALM provides eight models generated by eight approaches combing model selection method and criteria for entry/removal. The selecting of best models will be discussed in Results. Figure 2 provides a visual representation of the variables used in the ALM analysis.

## Results

### Descriptive Statistics

The descriptive statistics of the variables were computed on SPSS, Version 22. All the variables are normally distributed since their skewness values fall between −3 and +3 and kurtosis values between −10 and +10 (Kline, 2016). The correlation matrix indicates that generally the three groups of predictors, i.e., the eye-tracking, brain activation, and metacognitive measures, are not significantly related to each other.

### Model Fit Statistics Evaluation

As mentioned above, ALM generated eight models for the WLP and PLP tests, respectively. In ALM, models with smaller Information Criterion value fit better (IBM, 2018). As shown in Table 3, the “Best Subsets + Information Criterion (AICc)” model generated the lowest Information Criterion value (79.137) for the PLP test method; the “Forward Stepwise + Information Criterion (AICc)” and “Best Subsets + Information Criterion (AICc)” models generated the lowest value (71.867) for the WLP test method. The “Best Subsets + Information Criterion (AICc)” model also offers several advantages over the other models. Its model selection method, i.e., “Best Subsets,” can conduct “a computationally intensive search of the entire model space by considering all possible regression models of the pool of potential predictors” (Yang, 2013, p. 28). Besides that, “Best Subsets” is suggested for studies with 20 or fewer potential predictors (Miller, 2002; Yang, 2013). As for the criteria for entry/removal, “Information Criterion (AICc)” is suggested, because it is not prone to Type I and Type II errors and it works well for both small and large samples (Miller, 2002; Yang, 2013). Taken together, this study selected the model “Best Subsets + Information Criterion (AICc)” for both WLP and PLP test methods.

**TABLE 3:** Information criterion of different models in ALM.

No.	Models	Information Criterion	
		WLP	PLP
1.	Forward stepwise + Information Criterion (AICc)	71.867	79.165
2.	Forward stepwise + F statistics	71.885	81.767
3.	Forward stepwise + adjusted R^2^	72.836	80.461
4.	Forward stepwise + Overfit Prevention Criterion (ASE)	80.303	82.180
5.	Include all predictors	87.156	98.190
6.	Best subsets + Information Criterion (AICc)	71.867	79.137
7.	Best subsets + adjusted R^2^	72.836	80.461
8.	Best subsets + Overfit Prevention Criterion (ASE)	80.303	82.180

*AICc, Akaike’s Information Criterion with small-sample correction; ALM, automatic linear modeling; ASE, averaged square error.*

### Selected Model for the While-Listening Performance Tests

As shown in Table 4, 25.9% of the variance in the WLP test scores was significantly predicted by three variables (adjusted R^2^ = 0.259, *F*(4, 75) = 7.904, *p* = 0.000). Of these, person knowledge was the most important predictor (*B* = 0.467, *p* = 0.000), followed by fixation duration (*B* = −2.984, *p* = 0.002), and mental translation (*B* = 0.101, *p* = 0.027).

**TABLE 4:** Automatic linear modeling results for the WLP tests.

Variables (IV)	*B*	*Std. Error*	*t*-value	*F*-value	*p-*value	95% Confidence Interval		Importance
						Lower	Upper	
PK	0.467	0.116	4.046	16.373	0.000	0.237	0.698	0.483
WLP-NormAVFixDur	–2.984	0.932	–3.201	10.248	0.002	–4.842	–1.127	0.302
MT	0.101	0.045	2.260	5.106	0.027	0.012	0.190	0.151

*Adjusted R^2^ = 0.259. B = unstandardized coefficient. MT, Mental Translation; NormAVFixDur, normalized average fixation duration; PK, Person Knowledge; WLP, While-listening performance.*

### Selected Model for the Post-Listening Performance Tests

As shown in Table 5, 32.4% of the variance in the PLP test scores was significantly predicted by six variables (adjusted R^2^ = 0.324, *F*(7, 72) = 6.410, *p* = 0.000). Of these, dmPFC measure in the answering questions phase (PLP-Q-dmPFC) was the most important predictor (*B* = −5334.720, *p* = 0.000), followed by mental translation (*B* = 0.130, *p* = 0.006), directed attention (*B* = −0.481, *p* = 0.014), visit counts in the answering questions phase (PLP-Q-VisCounts) (*B* = 0.097, *p* = 0.027), IFG measure in the audio texts listening phase (PLP-A-IFG) (*B* = 5672.498, *p* = 0.046), and IFG measure in the answering questions phase (PLP-Q-IFG) (*B* = 2423.003, *p* = 0.048).

**TABLE 5:** Automatic linear modeling results for the PLP tests.

Variables (IV)	*B*	*Std. Error*	*t*-value	*F*-value	*p-*value	95% Confidence Interval		Importance
						Lower	Upper	
PLP-Q-dmPFC	−5334.720	1368.554	−3.898	15.195	0.000	−8062.881	−2606.558	0.325
MT	0.130	0.046	2.815	7.926	0.006	0.038	0.223	0.170
DA	−0.481	0.190	−2.524	6.371	0.014	−0.860	−0.101	0.136
PLP-Q-NormVisCounts	0.097	0.043	2.260	5.106	0.027	0.011	0.182	0.109
PLP-A-IFG	5672.498	2797.421	2.028	4.112	0.046	95.941	11249.054	0.088
PLP-Q-IFG	2423.003	1204.296	2.012	4.048	0.048	22.282	4823.723	0.087

*Adjusted R^2^ = 0.324. B = unstandardized coefficient. A, the audio texts listening phase; DA, directed attention; dmPFC, dorsal medial prefrontal cortex; IFG, inferior frontal gyrus; MT, mental translation; NormVisCounts, normalized visit counts; PLP, post-listening performance; Q, the answering questions phase.*

## Discussion

### Predictors of While-Listening Performance Test Performances

**FIGURE 3:**
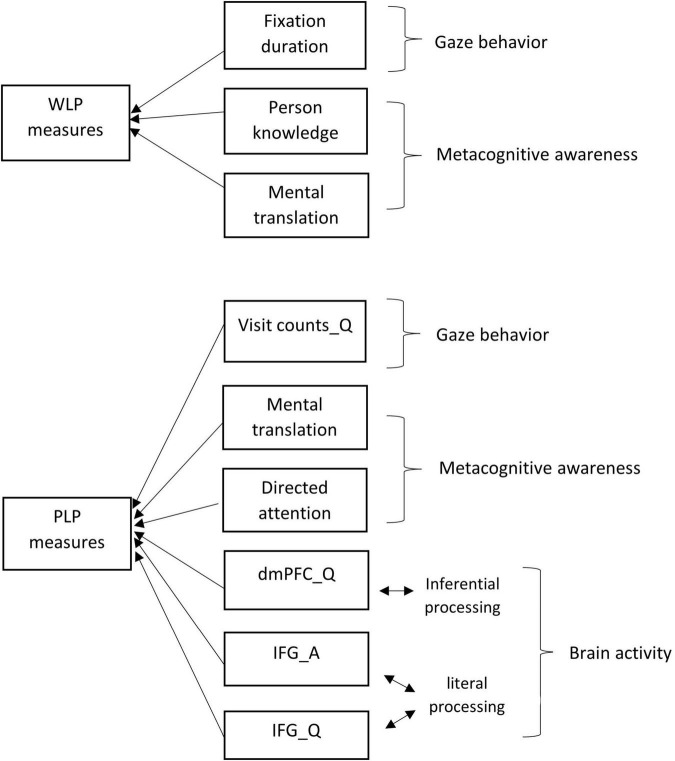
The visual representation of the ALM models for the WLP and PLP test methods. A, the audio texts listening phase; ALM, automatic linear modeling; dmPFC, dorsomedial prefrontal cortex; IFG, inferior frontal gyrus; PLP, post-listening performance; Q, the answering phase; WLP, while-listening performance.

ALM identified three listener-related factors significantly accounting for 25.9% of the variance in WLP test scores: (i) person knowledge and (ii) mental translation in metacognitive awareness, and (iii) fixation duration in gaze behaviors (Figure 3). The two metacognitive measures had positive relationships with WLP listening performance, whereas the gaze measure had a negative relationship.

Person knowledge concerns test takers’ self-appraisal as listeners, specifically their confidence and anxiety during listening. A higher person knowledge score indicates greater confidence and lower anxiety in listening (Vandergrift et al., 2006). The positive role of person knowledge in listening performance is unsurprising, because high confidence and low anxiety contribute to better listening performance, which is partially evidenced in Zhang’s (2013) study reporting a negative relationship between listeners’ anxiety level and listening performance. Confirmation of the predictive role of person knowledge in this study provides empirical support for theoretical considerations in listening, as anxiety is listed as an important affective person-factor both in Imhof and Janusik’s (2006) listening model and in Vandergrift and Goh’s (2012) systems model of listening. Notably, this finding is consistent with previous studies (Goh and Hu, 2014; Aryadoust, 2015; In’nami and Koizumi, 2022) that identified person knowledge as an important positive predictor of WLP listening performance.

The second predictor in the regression model was fixation duration, which showed a negative relationship with WLP listening performance. The verification of fixation duration as a significant negative predictor indicates that fixating on test items for a long time does not enable test takers to achieve higher scores. That is, long fixating gaze on test items during listening is a behavior signifying low listening ability. One possible reason is that the presence of test items in WLP tests in this study engaged test takers in multitasking, i.e., the simultaneous reading of test items, listening to the audio texts, and completing test items, which perhaps imposes extra cognitive load on listeners and distracts their attention from listening. Another reason might be that long fixating gaze indicates the use of “shallow listening.” When test takers fixate their gaze on test items, they are likely looking for cues in the test items, such as keywords and phrases, and then match them with those presented in the audio texts in order to locate possible answers. This is consistent with Field’s (2009) study that found WLP listeners were engaged in using keyword matching strategies. Field (2009) suggested that keyword-matching in the listening test condition was indicative of local-level processing, as test takers were neither able to recall the main topics of interest nor link the main points of the audio texts when asked in the retrospective reports. That is, WLP test takers tend to focus on lexical matches rather than generating a global representation of the audio texts based on the main points, resulting in shallow listening under test conditions. Field (2009) also suggested that shallow listening is a detriment to the comprehension of audio texts and hence would negatively affect listening test performance. Likewise, in the present study, the demand for intensive reading of—or gazing at, more precisely put—the test items in a listening test measured as fixation duration likely involved test takers in keyword matching and shallow listening, thereby adversely affecting their listening test performance. Furthermore, the use of test-wise strategies in listening tests would introduce construct-irrelevant variance into listening test scores representing the latent listening construct. The engagement in the attentive gaze fixation on test items while listening would also result in a considerable deviation of listeners’ cognitive processes and behaviors from those in listening activities of the target language usage (TLU) domain, thus minimizing the authenticity of the test (Aryadoust, 2019b).

The last significant predictor of WLP listening performance was mental translation, which is a subconstruct of metacognitive awareness in listening. Mental translation consists of the online strategies that listeners use to translate what they hear into their mother tongue. Due to the wording of the items (e.g., “I translate in my head as I listen”) and use of reverse coding, as suggested by previous studies (e.g., Goh and Hu, 2014), a lower mental translation score indicates more mental translation strategy use. Therefore, the positive relationship between mental translation scores and WLP listening scores indicates a functionally negative relationship between mental translation strategy use and listening scores. Mental translation often occurs when less-proficient listeners over-rely on literal listening processing, especially when their lexical, grammatical, and syntactic knowledge is deficient (Bonk, 2000; Goh and Hu, 2014). This finding is consistent with previous studies suggesting that L2 listeners should avoid using mental translation strategies to become more successful listeners (Vandergrift, 2003; Vandergrift et al., 2006; Vandergrift and Goh, 2012; Aryadoust, 2015; In’nami and Koizumi, 2022). We postulate that mental translation becomes a parasitic mental process in listening. That is, listeners allocate cognitive effort to conduct an “unnecessary” mental translation of the passage into their first language during listening. The cognitive activity of translation may tax the limited working memory of listeners and distract their attention to the lexis and sentences at the local level, hence resulting in inefficiency in generating a mental representation of the audio stimuli.

Notably, no neuroimaging measure was identified as a significant predictor of WLP listening performance. The absence of significant neuroimaging predictors indicates that test takers’ brain cortices supporting comprehension in both bottom-up and top-down fashions were not as significantly activated during the WLP test as other brain regions that subserve viewing and metacognition. These regions, which were not examined in the present study, include the visual cortex which subserves viewing and reading and the posterior region of the brain (Buchweitz et al., 2009). In addition, the dorsal anterior cingulate cortex (dACC) and lateral frontopolar cortex (lFPC) (Qiu et al., 2018) as well as the lateral prefrontal cortex (Fleming and Dolan, 2012) are known as the regulators of metacognitive thinking, which should be examined in future research.

Overall, the findings in this study provides evidence of sources of construct-irrelevant variance such as strategies and gaze in WLP listening tests, while relative to these factors, the brain regions subserving comprehension remained ineffective in predicting test performance. Consistent with previous research, this multifarious evidence shows the problematic nature of WLP tests and, therefore, the interpretations and uses of WLP test scores should be carried out with extreme caution.

### Predictors of Post-Listening Performance Test Performances

The prediction model for the PLP listening performance was different from that of WLP and consisted of a more complex network of predictors. 32.4% of the variance in PLP test scores was explained by six listener-related factors: (i) the dmPFC measure in PLP-Question, (ii) the IFG measure in PLP-Audio, and (iii) the IFG measure in PLP-Question of the brain measures, (iv) mental translation and (v) directed attention of the metacognitive measures, and (vi) visit counts in PLP-Question of the gaze measures (Figure 3). Of these, the dmPFC measure in PLP-Question and directed attention were negatively related to PLP test scores; the other predictors were positively related to the scores.

Unlike WLP tests, three brain variables were identified as significant predictors of PLP test scores: (i) dmPFC (ii) IFG in PLP-Question, and (iii) IFG in PLP-Audio, among which the first was negative and the rest were positive. In listening to audio texts, literal processing was dominant as indicated by the significant activation of the IFG. On average, unlike WLP test takers who were presented with test items while listening, PLP test takers would have no chance to search for cues in the test items to incorporate prior knowledges for inferences and predictions while listening. Instead, in order to comprehend the audio passages, PLP test takers would have to decode various pieces of information from phonemes to lexis and all the way to higher levels (e.g., discourse) in a bottom-up fashion (Field, 2004; Bodie et al., 2008). As the IFG is often involved in literal processing (e.g., Binder et al., 2009; Friederici, 2011; Buchweitz et al., 2014), it is plausible that the IFG subserved PLP test takers’ cognitive processing in this phase and played a positive role in their performance.

In the second phase of the PLP tests, where test takers answered questions with the help of their notes taken during listening, the dmPFC and IFG measures significantly predicted PLP test scores in a negative and positive manner, respectively. The results indicate that when answering the test items, the PLP test takers would have to rely on their notes by encoding the keywords of audio texts noted down (i.e., literal processing) more than making inferences based on their prior knowledge (i.e., inferential processing) to formulate proper answers. Therefore, higher activation of the IFG, which oversees literal processing, exerted a positive impact on test scores, while high involvement of the dmPFC, which is associated with inferential processing, would result in lower test scores. This contradicts previous studies that associated higher amounts of inferential processing with higher authenticity and optimal validity (e.g., Field, 2009) in listening. One reason for this contradiction might be that our neurophysiological design allowed for separating the listening process from the answering process, whereas in previous research these were never separated. In addition, previous claims (e.g., Field, 2009) were mostly speculative or relied heavily on test takers’ self-reports, which can be quite biased and imprecise. The results of this study should be extended in future research by comparing dmPFC activation in listening under test and non-test conditions and by setting clearer guidelines for interpreting inferential processing.

Additionally, two metacognitive awareness measures were verified to significantly predict the PLP performance: mental translation and directed attention. As such, the study is the first that supports the effect of these metacognitive strategies on PLP test performance. Like WLP, mental translation was also found to be positively related to PLP test scores, which indicates a negative relationship between the actual use of mental translation strategies and PLP listening scores due to the wording of the items and reverse coding. The similar findings for PLP and WLP tests suggest that mental translation strategy use should be minimized regardless of the test method if test takers aim for better listening performances.

The other metacognitive awareness variable was directed attention, which refers to the strategies that listeners use to concentrate and to stay on task. The negative relationship between directed attention and listening performance contrasts with Goh and Hu’s (2014) finding but is partially in line with Aryadoust’s (2015) finding that directed attention strategies were more commonly used by low-ability listeners. It could be that frequent use of directed attention strategies—i.e., focusing harder when having troubles understanding and not giving up when having difficulty understanding—would draw listeners’ attention toward local texts, such as unknown words or phrases. As an individual’s working memory capacity is limited (Wilhelm et al., 2013), allocating more cognitive effort to resolve local difficulties would interrupt the processing of the audio text, which may further cause information loss and the incoherence of the global representation of the auditory passage. Therefore, it is plausible to conclude that the use of directed attention strategy does not favor listening performance.

Another reason could be the role of lower directed attention in facilitating multitasking during listening tests, as evidenced in a previous study (Aryadoust, 2015). In the PLP-Audio phase, listeners are engaged in multitasking, as they need to take notes while listening due to the absence of test items and limited working memory capacity. Good coordination in such multitasking is conducive to better listening performance; without good coordination between notetaking and listening, listeners may either fail to jot down important notes due to the overwhelming incoming information from the audio texts or only achieve a superficial understanding because they are too busy with taking notes. Since low directed attention facilitates multitasking during listening (Aryadoust, 2015), it, in turn, supports listening test performance. This relationship could help explain the finding that low directed attention contributes to better PLP performance.

In terms of the eye-tracking measures, visit counts in the answering phase were identified as a significant positive predictor. This is likely because test takers needed to source information by switching their gazes back and forth between their notes on paper and the test items on the computer monitor (i.e., the area of interest (AOI) of the present study) when answering the PLP questions, which has resulted in a higher rate of visits inside and outside of the AOIs and hence higher visit counts. The frequent gaze switching also suggests PLP test takers’ greater reliance on their notes rather than inferences or even guesses when sourcing and formulating answers. This could be further evidence of why the IFG, the brain area associated with literal processing, played a significant role in the answering phase of the PLP tests as discussed above.

### Limitations of the Study

In this study, the listeners’ metacognitive awareness was measured by the MALQ, a questionnaire widely used in listening assessment. Nevertheless, this questionnaire is based on test takers’ self-reported responses, which makes the measurement of metacognitive awareness relatively subjective and subject to the reactivity effect (Double and Birney, 2019). Future studies should measure metacognitive awareness in a more objective manner, such as using eye-tracking and neuroimaging technology. Second, this study examined how test takers’ neurocognitive mechanisms affected listening test performance, but it did not explore whether individual differences, such as gender, nationality, and English as the first or second language, would affect the functioning of their neurocognitive mechanisms, including metacognitive strategy use. Future studies may include individual differences when investigating test takers’ neurocognitive mechanisms and metacognitive strategy use.

## Conclusion

To our knowledge, this is the first study that collectively examines listeners’ gaze behavior, brain activation, metacognitive awareness, and behavioral performance (i.e., test scores) under different test conditions to investigate the test method effects in listening assessment. Test takers’ listening performances were found to be significantly predicted by different listener-related factors under different test method conditions, indicating that different listening test methods activated different neurocognitive mechanisms of test taking. The comprehensive investigation of the listening construct from the behavioral, neurophysiological, and psychological perspectives contributes to a better understanding of it, especially of its multidimensional nature which changes as a function of test methods. This study also offers empirical evidence to support the role of metacognitive awareness in L2 listening to which educational practitioners are encouraged to attach importance. While the use of metacognitive strategies contributes to listening comprehension and the teaching of these strategies is recommended, the use of mental translation should, however, be avoided in listening teaching and testing, as this strategy may impede listening comprehension (Goh, 2002). The negative role of mental translation in predicting listening performance across test methods identified in the present study further supports this recommendation.

Nevertheless, overemphasizing the role of metacognitive awareness in listening is generally not recommended due to the relatively small proportion of variance that it accounts for in listening performance and its peripheral role in language proficiency, compared to the core components such as linguistic knowledge. We hope future research can extend this study and address its limitations to gain a deeper understanding of test method effects in listening assessment.

## Data Availability Statement

The datasets are available upon request from the data archive of Nanyang Technological University. Requests to access these datasets should be directed to VA, vahid.aryadoust@nie.edu.sg.

## Ethics Statement

The studies involving human participants were reviewed and approved by Nanyang Technological University. The patients/participants provided their written informed consent to participate in this study.

## Author Contributions

VA designed the study. JZ and VA examined and analyzed the data and reviewed and revised the manuscript. JZ wrote the first draft. All authors contributed to the article and approved the submitted version.

## Conflict of Interest

The authors declare that the research was conducted in the absence of any commercial or financial relationships that could be construed as a potential conflict of interest.

## Publisher’s Note

All claims expressed in this article are solely those of the authors and do not necessarily represent those of their affiliated organizations, or those of the publisher, the editors and the reviewers. Any product that may be evaluated in this article, or claim that may be made by its manufacturer, is not guaranteed or endorsed by the publisher.
